# Psychiatric Intensive Care Unit (Tryweryn Ward, North Wales) Performance Data and the Impact of Environmental Design on Restrictive Practices: A Comparative Analysis in North Wales

**DOI:** 10.1192/bjo.2026.11592

**Published:** 2026-06-30

**Authors:** Saima Mustafa, Rajvinder S Sambhi, Anna Walsh

**Affiliations:** 1 Betsi Cadwaladr University Health Board, Bodelwyddan, United Kingdom; 2 Betsi Cadwaladr University Health Board, Wrexham, United Kingdom

## Abstract

**Aims:**

This study aimed to quantify the clinical performance of the Tryweryn ward (PICU) in Heddfan Psychiatric Unit, Wrexham, North Wales against the data available nationally in the 2021 National Survey of PICUs. Our objective was also to evaluate how unit infrastructure; specifically, the presence or absence of a dedicated on-unit seclusion suite affects the frequency of restrictive physical interventions (RPI) in the two PICUs in North Wales (Tryweryn ward with no seclusion suite and Taliesin ward with a dedicated seclusion suite).

**Methods:**

A retrospective analysis was conducted on admission data over an 18-month period (N=190) at Tryweryn ward (PICU) in Wrexham. Data collected was on gender, legal status, diagnosis, and length of stay (LoS) and discharge/transfer destination. These were compared to nationally available data in previously published literature from PICUs in England. Additionally, a 13-month data (August 2024–August 2025) compared the number of restrictive physical intervention episodes in Tryweryn ward PICU with no seclusion suite versus such episodes in Taliesin Ward (PICU with dedicated seclusion suite).

**Results:**

Tryweryn Ward PICU data demonstrated high alignment with nationally available data for mean LoS (36.55 days vs. national 36.57 days) and primary diagnosis, with 58% of patients admitted in psychotic crisis. Gender distribution showed a higher female representation (37%) compared to the 20% national average. Legal acuity was high, with 88% of patients detained under Section 2 or 3 of the Mental Health Act.

Comparative data on restrictive practices revealed a significant disparity:

- **Seclusion**: The PICU with dedicated seclusion suite (Taliesin Ward) recorded 22 such episodes, whereas the unit without (Tryweryn) recorded only 2, utilizing off-unit Section 136 suites or transfers out of the unit for such a need.
- **RPI**: The ward with seclusion suite recorded 352 total RPI incidents versus Tryweryn ward incidents (139 incidents).
- **Monthly Variation**: In the final month of the data collection, RPI incident number peaked at 82 incidents in the PICU with seclusion, compared to 7 in the PICU without.

**Conclusion:**

Our PICU with no seclusion suite facility in North Wales effectively mirrors the nationally available data regarding intensive care psychiatric practice. The utility of seclusion suites remains debatable on the issue whether such suites contribute to any reduction in restrictive physical intervention practices or not.

